# Divergent effect of fast- and slow-releasing H_2_S donors on boar spermatozoa under oxidative stress

**DOI:** 10.1038/s41598-020-63489-4

**Published:** 2020-04-16

**Authors:** Eliana Pintus, Marija Jovičić, Martin Kadlec, José Luis Ros-Santaella

**Affiliations:** Department of Veterinary Sciences, Faculty of Agrobiology, Food and Natural Resources, Czech University of Life Sciences Prague, Kamýcká 129, 165 00 Praha, 6-Suchdol Czech Republic

**Keywords:** Cell biology, Reproductive biology

## Abstract

Hydrogen sulphide (H_2_S) is involved in the physiology and pathophysiology of different cell types, but little is known about its role in sperm cells. Because of its reducing properties, we hypothesise that H_2_S protects spermatozoa against the deleterious effects of oxidative stress, a condition that is common to several male fertility disorders. This study aimed i) to determine the total antioxidant capacities of Na_2_S and GYY4137, which are fast- and slow-releasing H_2_S donors, respectively, and ii) to test whether H_2_S donors are able to protect spermatozoa against oxidative stress. We found that Na_2_S and GYY4137 show different antioxidant properties, with the total antioxidant capacity of Na_2_S being mostly unstable and even undetectable at 150 µM. Moreover, both H_2_S donors preserve sperm motility and reduce acrosome loss, although the effects were both dose and donor dependent. Within the range of concentrations tested (3–300 µM), GYY4137 showed positive effects on sperm motility, whereas Na_2_S was beneficial at the lowest concentration but detrimental at the highest. Our findings show that Na_2_S and GYY4137 have different antioxidant properties and suggest that both H_2_S donors might be used as *in vitro* therapeutic agents against oxidative stress in sperm cells, although the optimal therapeutic range differs between the compounds.

## Introduction

Hydrogen sulphide (H_2_S) is the most recently discovered gaseous molecule that participates in a variety of biological functions, as do nitric oxide (NO) and carbon monoxide (CO). In mammals, H_2_S can be synthesised by enzymatic or non-enzymatic pathways^1^. Overall, it seems likely that most of the H_2_S produced within an organism is generated by the H_2_S-synthesising enzymes: cystathionine γ-lyase (CSE), cystathionine β-synthase (CBS), and 3-mercaptopyruvate sulphurtransferase (3-MST), with the latter coupled with cysteine aminotransferase (CAT)^2^.

In the male reproductive system, the expression of H_2_S-generating enzymes has been reported in the testis^3,4^, epididymis^5^, penile corpus cavernosum^6^, and spermatozoa^7,8^, which strongly suggests that this gasotransmitter is involved in sperm physiology to some extent. In a recent study, Wang *et al*. found that asthenospermic men show reduced levels of H_2_S in their seminal plasma and that exogenous H_2_S supplementation improves their sperm motility^8^. In contrast, in boar spermatozoa, H_2_S exerts no or negative effects on sperm motility, viability, and mitochondrial membrane potential^9^. With both positive and negative effects documented, there is still controversy concerning the role of H_2_S in sperm cells. This apparent discrepancy might, at least partly, be a result of H_2_S dose- and donor-dependent effects^10^.

According to their chemical structure and source, H_2_S donors include inorganic salts and derivatives of phosphorodithioate, garlic extracts, thioaminoacids, and anti-inflammatory drugs^11^. On the basis of their release mechanism, H_2_S donors can be classified in two categories: slow- and fast-releasing agents. Among the fast-releasing H_2_S donors, the inorganic salts sodium sulphide (Na_2_S) and sodium hydrosulphide (NaHS) are probably most frequently employed in biological studies. Both salts can be dissolved in aqueous solution, leading to an instantaneous release of H_2_S that mimics a bolus administration. Despite the common use of these donors in experimental studies, it is becoming increasingly clear that their gas release might not be representative of the physiological H_2_S levels in tissues and cells^12^. On the other hand, slow-releasing H_2_S donors, like the phosphorodithioate derivative GYY4137, produce a slow and continuous release of gas, which is more similar to the physiological conditions found within organisms^12^. For this reason, the use of different H_2_S donors in studies is useful to elucidate the biological activity and possible therapeutic effects^12^.

By virtue of its activity as a reducing agent, H_2_S attenuates the damage induced by oxidative stress in different cells and tissues (e.g. neurons^13^, gastric cells^14^, lung cells^15^). Oxidative stress is an underlying condition common to several male reproductive disorders, in which high levels of reactive oxygen species (ROS) cause sperm dysfunction (e.g. decreased sperm motility, impaired membrane and DNA integrity, increased lipid peroxidation) and infertility^16,17^. Previous studies have shown that H_2_S is able to alleviate the effects of oxidative stress on testicular functions^4,8,18^, but the *in vitro* effects of this gasotransmitter on sperm cells under a ROS-generating system still need to be elucidated.

The aim of this study was to evaluate the total antioxidant capacity and stability of the H_2_S donors Na_2_S and GYY4137 under standard conditions (38 °C, pH≈7) and at different times (i.e. 20, 120, and 210 minutes) during the incubation (experiment I). Because sperm motility under a ROS-generating system may drop in a few hours^19,20^ and based on the opposite modalities of H_2_S release by Na_2_S and GYY4137 (i.e. fast and slow release, respectively), these incubation times were chosen to determine the dynamics of the antioxidant activity of each donor during the early, mid, and late stages of incubation. Based on the results from experiment I and the physiological total antioxidant capacity of boar seminal plasma^21^, we then established a suitable range of concentrations of Na_2_S and GYY4137 to be tested in boar sperm samples under a ROS-generating system (experiment II). Although some Na_2_S and GYY4137 concentrations used in experiment II show a total antioxidant capacity that is below the range of detection by spectrophotometry, they were included in our experimental design because increasing evidence suggests that *in vivo* H_2_S levels range from low µM to high nM^22^. Next, we evaluated the effects of both donors on sperm motility, mitochondrial activity, plasma membrane integrity, acrosomal status, and lipid peroxidation. The results from this study elucidate the role of H_2_S donors in sperm samples under oxidative stress and the possible therapeutic implications of these compounds for alleviating the negative effects of ROS on sperm function.

## Results

### Experiment I. Total antioxidant capacity and stability of H_2_S donors

**Figure 1:**
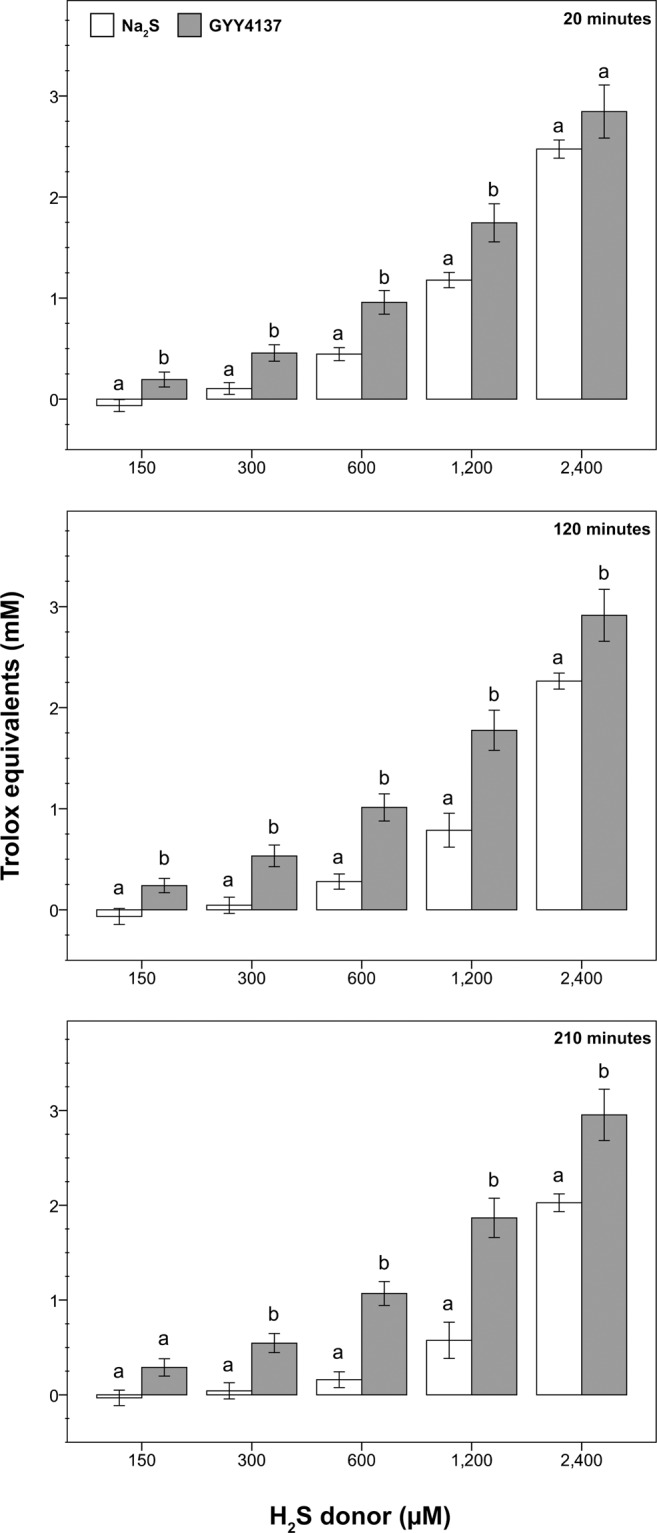
Total antioxidant capacity and stability of the H_2_S donors Na_2_S and GYY4137. The H_2_S donors were incubated in phosphate-buffered saline solution at 38 °C in a water bath. Total antioxidant capacity is expressed as Trolox equivalents (mM). White histograms: Na_2_S; grey histograms: GYY4137. Upper panel: 20 minutes of incubation; middle panel: 120 minutes of incubation; lower panel: 210 minutes of incubation. Different letters indicate significant differences (*p* < 0.05) between H_2_S donors at the same concentration and incubation time. Data are shown as the mean±standard error of four replicates.

As can be seen from Table 1, Na_2_S and GYY4137 showed different total antioxidant capacities and stabilities during the incubation. Overall, the total antioxidant capacity of Na_2_S significantly decreased between 20 and 210 minutes of incubation, whereas that of GYY4137 tended to increase during this period and was significantly higher after 210 minutes than after 20 minutes of incubation at 2,400 and 1,200 µM (*p* < 0.05). Moreover, GYY4137 showed detectable levels of total antioxidant capacity at all concentrations tested, whereas Na_2_S was unstable within the range of 300 to 1,200 µM and was undetectable at 150 µM. Irrespective of the concentration considered, GYY4137 showed greater total antioxidant capacity than Na_2_S (Fig. 1).

**Table 1:** Total antioxidant capacity and stability of the H_2_S donors Na_2_S and GYY4137.

Treatment	Concentration (µM)	Time (min)		
		20	120	210
Na_2_S	2,400	2,474.6 ± 89.8^a^	2,262.3 ± 79.4^ab^	2,027.1 ± 92.8^b^
	1,200	1,178.1 ± 75.7^a^	787.4 ± 168.1^ab^	575.2 ± 191.3^b^
	600	445.4 ± 64.6^a^	278.6 ± 75.1^ab^	160.2 ± 83.3^b^
	300	105.5 ± 58.0^a^	44.5 ± 80.1^a^	42.6 ± 85.2^a^
	150	n.d.	n.d.	n.d.
GYY4137	2,400	2,845.9 ± 262.7^a^	2,913.8 ± 257.3^ab^	2,954.2 ± 270.7^b^
	1,200	1,745.0 ± 188.8^a^	1,775.9 ± 199.1^ab^	1,867.2 ± 207.3^b^
	600	958.3 ± 117.2^a^	1,012.7 ± 135.0^a^	1,069.6 ± 125.3^a^
	300	456.0 ± 81.6^a^	532.8 ± 107.2^a^	546.0 ± 101.1^a^
	150	194.1 ± 73.7^a^	239.5 ± 70.5^a^	289.4 ± 91.3^a^
PBS		n.d.	n.d.	n.d.

The H_2_S donors were incubated in phosphate-buffered saline solution at 38 °C in a water bath. Total antioxidant capacity is expressed as Trolox equivalents (µM). Different superscripts indicate significant differences (p < 0.05) among times within each donor concentration. PBS: phosphate-buffered saline solution; n.d.: not detectable. Data are shown as the mean±standard error of four replicates.

### Experiment II. Effect of H_2_S donors on boar sperm parameters under induced oxidative stress

#### Sperm motility

**Figure 2:**
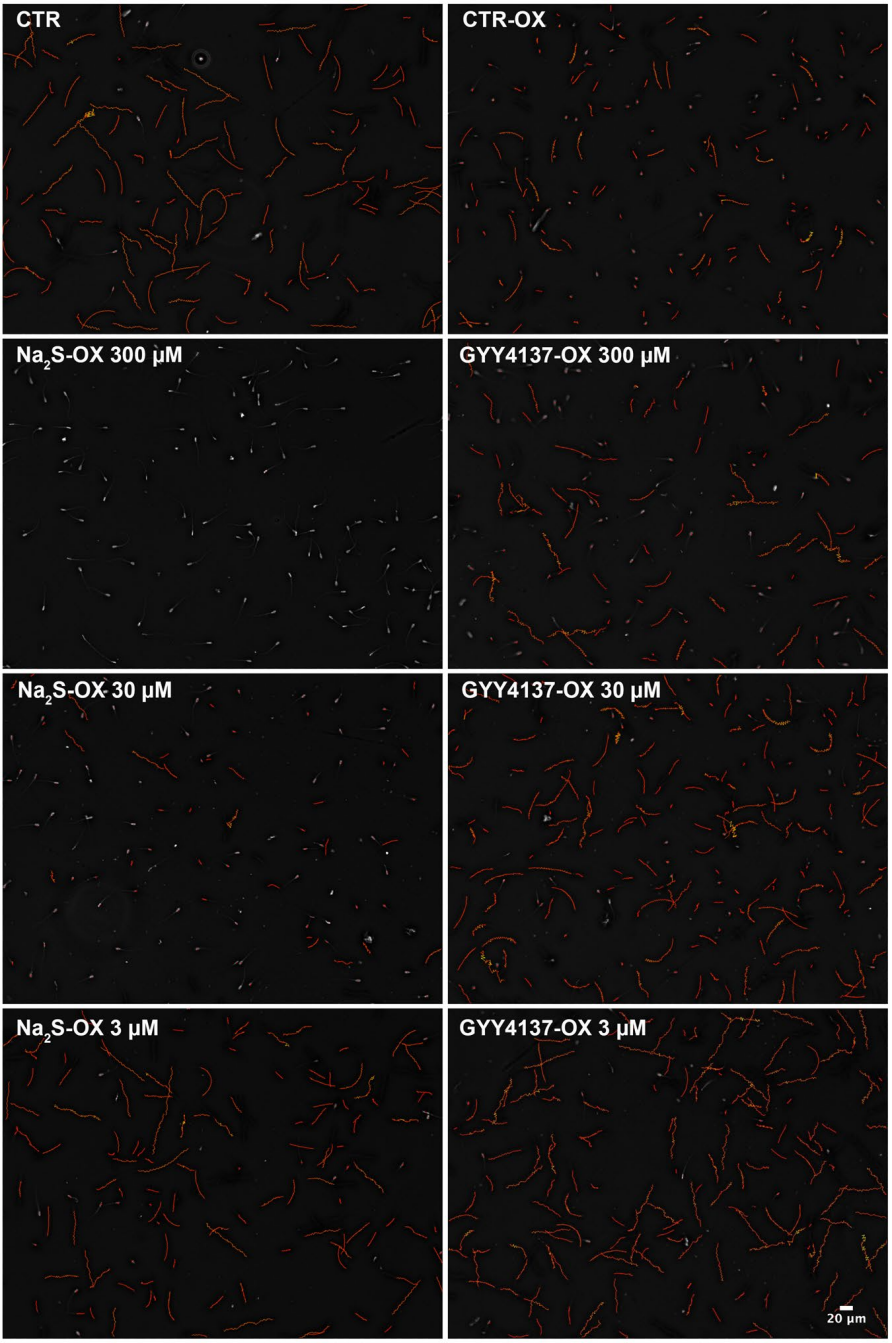
Boar sperm motility in samples submitted to oxidative stress and supplemented with the H_2_S donors Na_2_S and GYY4137. Representative images of sperm trajectories assessed by Computer-Assisted Sperm Analysis (CASA). Red trajectories show motile spermatozoa (cells are not shown because of overlapping with the trajectories), whereas immotile sperm cells are fully shown.

Overall, the effects of H_2_S on boar sperm motility under induced oxidative stress were dose- and donor-dependent (Table 2, Fig. 2). Thus, 3 and 30 µM GYY4137 and 3 µM Na_2_S preserved the sperm motility and kinetics under the ROS-generating system. Interestingly, all of the sperm kinetic parameters in these treatments did not differ from those of the control group without oxidative stress (CTR; *p* > 0.05). The results with both 3 and 30 µM GYY4137 showed higher percentages of total motility (TM) relative to that of the control group under oxidative stress (CTR-ox; *p* < 0.01). Moreover, a dose of 30 µM GYY4137 significantly increased the percentage of progressive motility (PM) over that in the CTR-ox group (*p* = 0.040). Although differences were not statistically significant, higher average path velocity (VAP) and straight-line velocity (VSL) were observed in samples treated with 3 µM GYY4137 than in those in the CTR-ox group (*p* = 0.071 and *p* = 0.064, respectively). On the other hand, the effects of Na_2_S were markedly dose dependent. At 300 µM, this fast-releasing H_2_S donor showed clear negative effects on sperm motility. No motile spermatozoa were observed in any replicate; therefore, no kinetics data could be provided. By contrast, at the lowest concentration, Na_2_S significantly increased the percentage of motile sperm cells relative to that in the CTR-ox group (*p* = 0.018). At a concentration of 30 µM, Na_2_S greatly decreased the TM, PM, VAP, and VSL (*p* < 0.05), although it did not affect the curvilinear velocity (VCL) and the remaining motion parameters in comparison with those of the CTR-ox group (*p *> 0.05). However, at this Na_2_S concentration, we observed some variability among the replicates with the percentage of motile spermatozoa ranging from 0 to almost 30%. There were no differences between the CTR-ox and H_2_S donor treatments in the amplitude of lateral head displacement (ALH), beat-cross frequency (BCF), linearity (LIN), straightness (STR), and wobble (WOB; *p* > 0.05).

**Table 2:** Boar sperm motility and kinetics in samples submitted to oxidative stress and supplemented with the H_2_S donors Na_2_S and GYY4137.

Treatment	Conc. (µM)	Time (min)	TM (%)	PM (%)	VAP (µm/s)	VCL (µm/s)	VSL (µm/s)	ALH (µm)	BCF (Hz)	LIN (%)	STR (%)	WOB (%)
CTR		20	75.6 ± 2.9	52.8 ± 5.8	43.1 ± 1.7	83.8 ± 3.7	34.8 ± 1.6	3.1 ± 0.1	13.5 ± 0.4	41.7 ± 2.8	80.7 ± 2.8	50.1 ± 1.9
CTR		210	73.1 ± 3.4^a^	70.8 ± 5.2^ac^	42.0 ± 2.6^a^	69.0 ± 5.1^a^	37.8 ± 1.8^a^	3.1 ± 0.6^a^	15.6 ± 0.6^a^	55.8 ± 3.0^a^	89.9 ± 2.3^a^	61.0 ± 2.3^a^
CTR-ox		210	46.7 ± 8.7^c^	65.8 ± 2.3^a^	33.1 ± 5.4^b^	52.7 ± 9.6^ab^	30.8 ± 4.7^a^	2.1 ± 0.4^bc^	16.3 ± 0.5^a^	62.9 ± 2.9^a^	93.5 ± 1.2^a^	66.4 ± 2.5^a^
Na_2_S-ox	300	210	n.a.	n.a.	n.a.	n.a.	n.a.	n.a.	n.a.	n.a.	n.a.	n.a.
	30	210	7.9 ± 5.5^d^	44.8 ± 5.5^b^	21.7 ± 2.0^c^	34.7 ± 2.7^b^	20.3 ± 2.1^b^	1.5 ± 0.1^c^	15.1 ± 0.5^a^	62.3 ± 1.6^a^	94.3 ± 0.7^a^	65.6 ± 1.3^a^
	3	210	65.6 ± 6.2^ab^	69.3 ± 5.1^ac^	39.2 ± 2.6^ab^	64.7 ± 8.0^a^	35.3 ± 1.6^a^	2.6 ± 0.2^ab^	15.7 ± 0.5^a^	58.1 ± 4.4^a^	90.6 ± 2.8^a^	63.0 ± 3.3^a^
GYY4137-ox	300	210	57.2 ± 6.9^bc^	70.5 ± 3.4^ac^	35.5 ± 4.4^ab^	56.0 ± 8.2^a^	33.0 ± 3.8^a^	2.3 ± 0.3^abc^	16.0 ± 0.3^a^	61.8 ± 3.0^a^	93.0 ± 1.6^a^	65.6 ± 2.4^a^
	30	210	69.0 ± 6.2^ab^	77.2 ± 2.5^c^	38.1 ± 3.7^ab^	59.0 ± 6.7^a^	35.6 ± 3.3^a^	2.5 ± 0.3^ab^	16.2 ± 0.4^a^	62.2 ± 2.8^a^	93.0 ± 1.3^a^	66.2 ± 2.2^a^
	3	210	69.8 ± 7.7^ab^	75.3 ± 3.7^ac^	41.3 ± 4.0^ab^	65.9 ± 7.5^a^	38.0 ± 3.5^a^	2.7 ± 0.3^ab^	16.1 ± 0.5^a^	59.3 ± 2.8^a^	91.8 ± 1.5^a^	63.8 ± 2.2^a^

Different superscripts within the same column indicate significant differences (p < 0.05) among treatments within the same incubation time. Conc.: concentration; TM: total motility; PM: progressive motility; VAP: average path velocity; VCL: curvilinear velocity; VSL: straight-line velocity; ALH: amplitude of lateral head displacement; BCF: beat-cross frequency; LIN: linearity (VSL/VCL); STR: straightness (VSL/VAP); WOB: wobble (VAP/VCL); CTR: control; ox: samples submitted to induced oxidative stress; n.a.: not available. Data are shown as the mean±standard error of six replicates.

#### Sperm mitochondrial status

**Figure 3:**
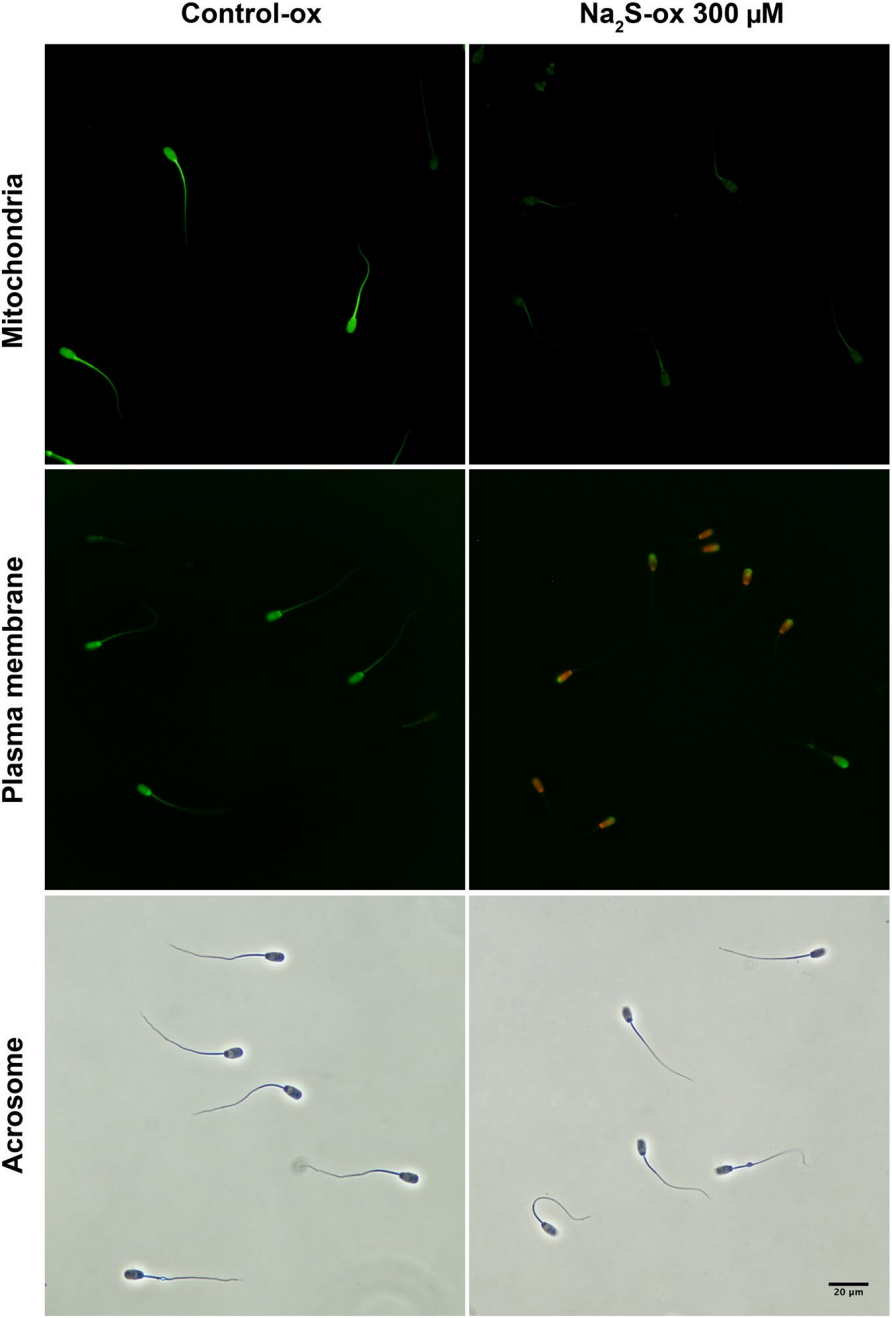
Detrimental effects of a high concentration of Na_2_S, a fast-releasing H_2_S donor, on the boar sperm mitochondrial status and the plasma membrane and acrosome integrities (normal apical ridge) under oxidative stress. Representative images of sperm cells assessed by epifluorescence microscopy (mitochondrial status was assessed by using rhodamine 123 and propidium iodide; plasma membrane integrity was assessed by using carboxyfluorescein diacetate and propidium iodide) or phase-contrast microscopy (the normal apical ridge was assessed after fixation with glutaraldehyde).

At a concentration of 300 µM, Na_2_S showed clear negative effects on boar sperm mitochondrial status. In every replicate, there were no spermatozoa with active mitochondria (Table 3, Fig. 3). There were no differences between the CTR-ox group and the remaining treatment groups (*p* > 0.05).

**Table 3:** Boar sperm mitochondrial status, plasma membrane integrity, and acrosomal status in samples submitted to oxidative stress and supplemented with the H_2_S donors Na_2_S and GYY4137.

Treatment	Conc. (µM)	Time (min)	Active mitochondria (%)	Intact plasma membrane (%)	Intact acrosome (NAR, %)	Acrosome loss (PNA, %)
CTR		20	56.0 ± 3.2	82.0 ± 2.2	95.1 ± 0.9	1.4 ± 0.3
CTR		210	62.8 ± 3.3^a^	76.6 ± 2.3^a^	94.8 ± 1.0^a^	2.4 ± 0.2^a^
CTR-ox		210	62.4 ± 1.2^a^	67.8 ± 3.9^abc^	92.9 ± 0.8^a^	3.9 ± 0.7^b^
Na_2_S-ox	300	210	0^b^	21.2 ± 6.0^d^	31.1 ± 5.6^b^	2.0 ± 0.3^a^
	30	210	60.8 ± 5.1^a^	66.1 ± 4.7^bc^	94.6 ± 0.7^a^	2.2 ± 0.4^a^
	3	210	62.1 ± 3.3^a^	74.8 ± 3.3^abc^	92.8 ± 0.6^a^	1.7 ± 0.4^a^
GYY4137-ox	300	210	67.7 ± 2.8^a^	70.8 ± 3.8^abc^	93.0 ± 1.4^a^	1.5 ± 0.4^a^
	30	210	62.9 ± 2.9^a^	75.8 ± 3.2^a^	94.1 ± 0.9^a^	2.3 ± 0.4^a^
	3	210	63.1 ± 1.8^a^	76.5 ± 3.6^a^	94.2 ± 0.6^a^	1.8 ± 0.4^a^

Different superscripts within the same column indicate significant differences (*p *< 0.05) among treatments within the same incubation time. Conc.: concentration; NAR: normal apical ridge; PNA: peanut agglutinin-fluorescein isothiocyanate; CTR: control; ox: samples submitted to induced oxidative stress. Data are shown as the mean±standard error of six replicates.

#### Sperm plasma membrane integrity and lipid peroxidation

**Figure 4:**
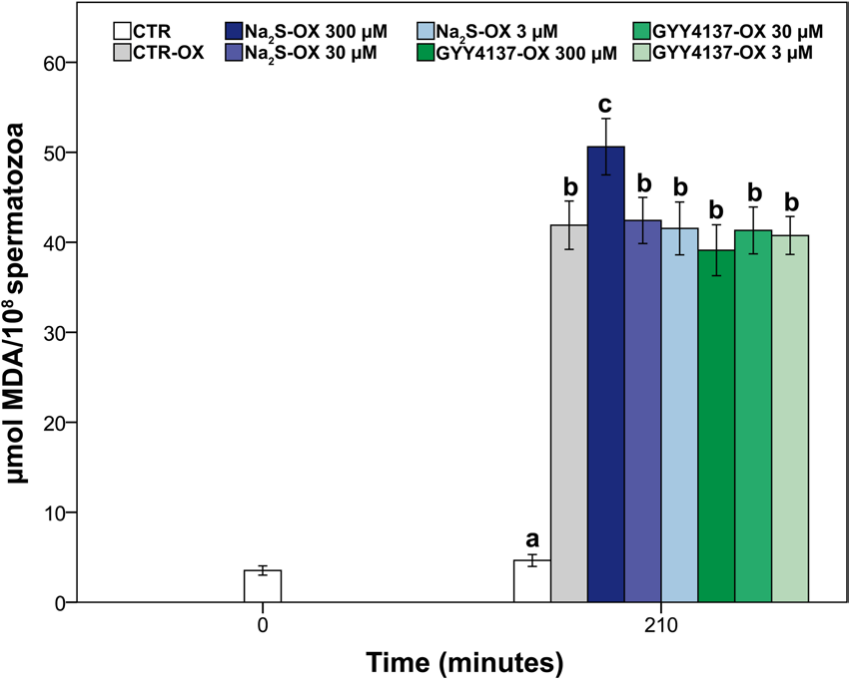
Lipid peroxidation in boar sperm samples submitted to oxidative stress and supplemented with the H_2_S donors Na_2_S and GYY4137. CTR: control; ox: samples submitted to induced oxidative stress; MDA: malondialdehyde. Different letters indicate significant differences (*p* < 0.05) among treatments. Data are shown as the mean±standard error of six replicates.

There was no significant effect of GYY4137 on sperm plasma membrane integrity at any of the concentrations used (*p* > 0.05, Table 3). On the other hand, 300 µM Na_2_S markedly impaired the plasma membrane integrity relative to the results obtained for the CTR-ox group (*p* < 0.001, Table 3, Fig. 3). No effects were observed at the remaining Na_2_S concentrations (*p* > 0.05). A similar pattern was observed for the sperm lipid peroxidation: higher values of malondialdehyde (MDA) per 10^8^ spermatozoa were observed in samples treated with 300 µM Na_2_S than those in the CTR-ox group and the other treatment groups (*p* < 0.01, Fig. 4). No differences in the MDA levels were found between the CTR-ox group and the remaining treatment groups (*p* > 0.05).

#### Acrosomal status

We found that 300 µM Na_2_S impaired the acrosome integrity (normal apical ridge or NAR test) relative to that of the CTR group, both with and without oxidative stress (*p* < 0.001, Table 3, Fig. 3). No differences were observed in the NAR test results between the CTR-ox group and the other treatment groups (*p* > 0.05). On the other hand, all treatments showed lower percentages of acrosome-lost spermatozoa (evaluated with peanut agglutinin–fluorescein isothiocyanate, PNA-FITC) than that of the CTR-ox group (*p* ≤ 0.001, Table 3).

## Discussion

In this study, we provide the first evidence, to the best of our knowledge, that Na_2_S and GYY4137 show different total antioxidant capacities and stabilities under standard conditions (38 °C, physiological pH) and after different periods (20, 120, and 210 minutes) of incubation. Our results also reveal that the total antioxidant capacity of Na_2_S is less stable than that of GYY4137, although the latter’s total antioxidant capacity tends to increase over time. This phenomenon should be taken into account in studies entailing cell incubation at 38 °C and at physiological pH, because the release of H_2_S by GYY4137 is both pH and temperature dependent^12^. Moreover, GYY4137 shows higher total antioxidant capacity than that of Na_2_S after any incubation time, with the total antioxidant capacity of Na_2_S at 150 µM even being undetectable by spectrophotometry after 20 minutes of incubation. The patterns observed in the antioxidant capacities of these H_2_S donors may not reflect their H_2_S release, given that the inorganic salts Na_2_S and NaHS lead to a larger but shorter gas release than GYY4137^23,24^.

Our results show that both H_2_S donors partly palliate the damages provoked by oxidative stress in sperm cells, supporting the results found previously in other cells (neurons^13,25^, gastric cells^14^, testicular germ cells^4^) and organs (lungs^15,26^, testes^18^). In these studies, oxidative stress was induced by *in vivo* or *in vitro* ROS-generating systems, such as glutamate, hydrogen peroxide (H_2_O_2_), heat, tobacco smoke, ovalbumin sensitisation, and ischaemia-reperfusion injury. In our study, we used Fe^2+^/ascorbate, which induces lipid peroxidation and catalyses the production of hydroxyl radicals (^•^OH), the most powerful free radical known, by the Fenton reaction^27^. Moreover, several previous studies^4,13–15,18,25,26^ employed a single H_2_S donor, whereas two H_2_S-releasing agents were tested in our study. To date, few studies^8,23,28^ have investigated the biological effects of both fast- and slow-releasing H_2_S donors in cells under oxidative stress conditions. In agreement with these studies, our findings confirm that the effects of H_2_S donors are not only dose but also donor dependent. Moreover, for the first time to the best of our knowledge, the *in vitro* effects of fast- and slow-releasing H_2_S donors were evaluated in sperm cells in the presence of a ROS-generating system. Overall, we found that the slow-releasing H_2_S donor GYY4137 did not show any cytotoxic effect. Moreover, 3 and 30 µM GYY4137 preserved the boar sperm motility against the detrimental effects of oxidative stress. Interestingly, at both concentrations, the percentage of motile sperm cells was almost 50% higher than that of the CTR-ox samples and no kinetic parameters differed from those of the CTR group without oxidative stress. Moreover, 30 µM GYY4137 samples showed a higher percentage of progressive motility than those in the CTR-ox group. However, in contrast to other studies^8,18^, we did not find any effect of GYY4137 on the levels of lipid peroxidation, which may be due to the different cell type and donor concentration used in our study. Our findings also show that, in comparison with GYY4137, the effects of Na_2_S were markedly dose dependent. At a concentration of 3 µM, this fast-releasing H_2_S donor preserves boar sperm motility (40.5% higher than that in the CTR-ox samples), whereas it shows clear cytotoxic effects at 300 µM. This pattern may reflect the well-known biphasic biological dose–response curve of H_2_S: it acts as an antioxidant at low concentrations, but a pro-oxidant at high concentrations^29^. Interestingly, we also found that 30 µM Na_2_S was not cytotoxic (i.e. no effect was observed on the mitochondrial activity, plasma membrane integrity, acrosome integrity, or lipid peroxidation), but it strongly inhibited sperm motility. As in our findings, Zhao *et al*. found that 25 µM Na_2_S inhibits boar sperm motility although it does not affect the viability and mitochondrial membrane potential^9^. Likewise, NaHS has been shown to inhibit the motility of mouse and human spermatozoa^8,30^. In our study, the inhibitory effects of Na_2_S on sperm motility were described by using a comprehensive set of kinetic parameters provided by Computer-Assisted Sperm Analysis (CASA). Overall, 30 µM Na_2_S remarkably decreased the sperm motility, VAP, and VSL, but it did not affect the remaining motion parameters. In spite of some variability among replicates, a small percentage of sperm cells showed very slow but progressive movement. With regard to the mechanism of action, Zhao *et al*. found that the inhibitory effects of Na_2_S on sperm motility are related to the adenosine 5′-monophosphate-activated protein kinase (AMPK) and protein kinase B (AKT) pathways^9^, whereas a more recent study revealed that H_2_S promotes the secretion of K^+^ in the epididymis; this suppresses sperm motility and may contribute to keeping the sperm cells in a quiescent state before ejaculation^5^. Another plausible explanation for the reduced sperm motility elicited by Na_2_S can be provided by the inhibitory effect of H_2_S on cytochrome *c* oxidase (complex IV), the final component of the electron transport chain that plays a key role in aerobic respiration and adenosine triphosphate (ATP) generation^29^. Because mammalian spermatozoa devote most of the energy generated as intracellular ATP to motility^31^, a reduction in ATP levels would lead to an immediate decrease in sperm motility. In this way, for instance, cardiac cells treated with 25 µM Na_2_S showed over 80% decreased O_2_ consumption rate relative to the baseline^32^. In contrast to the effects observed at low concentrations, high concentrations of Na_2_S showed clear detrimental effects: no motility or active mitochondria were observed in any sample treated with 300 µM Na_2_S. The toxic effects were acute and led to immediate failure of sperm motility and mitochondrial activity (personal observations). This phenomenon can be explained by the high levels of ROS induced by Na_2_S^9^, an explanation corroborated by the increased levels of lipid peroxidation and the low percentage of sperm with intact plasma membrane and acrosome (NAR) found in our study. In contrast to our findings, in testicular germ cells, Li *et al*. found that treatment with NaHS in the concentration range of 1–200 µM for 30 minutes does not affect cell viability, although cell injuries are induced at 1 mM^4^. This result might be because, unlike other male germ cells, spermatozoa have limited antioxidant defences. Spermatozoa lack the necessary cytoplasmic-enzyme repair systems, and their membranes are particularly rich in polyunsaturated fatty acids, which make these cells particularly susceptible to the damage caused by oxidative stress^16^. Another reason might be related to the use of open systems (e.g. four-well plates and Petri dishes) that lead to quick volatilisation of the gas during cell incubation^33^. It is known that Na_2_S volatilises very quickly^33^, and the use of closed systems, such as the one used in our study, delays this process and leads to more consistent results regarding the effects of H_2_S donors on cell biology. Our results also show that Na_2_S and GYY4137 partly reduce the damage induced by high ROS levels on acrosomal status by decreasing the percentage of acrosome-lost (PNA-FITC) spermatozoa relative to that in the CTR-ox group. However, neither of the two donors showed any positive effect on the acrosome integrity evaluated by the NAR test. This divergence could be explained by the different acrosomal attributes considered by these two methodologies: NAR evaluated in unstained samples by phase-contrast microscopy versus outer acrosomal membrane integrity assessed by PNA-FITC staining with epifluorescence microscopy^34,35^. Because acrosome integrity is a requisite for fertilisation and the ROS levels affect sperm function^36,37^, it remains to be tested whether Na_2_S and GYY4137 may preserve the fertilising potential of sperm cells under oxidative stress. Semen handling and storage decrease boar sperm quality and fertilising ability, probably because of oxidative stress^38,39^, so H_2_S donors may be useful for the optimisation of semen extenders used in artificial insemination programmes. In the porcine industry, higher efficiency of artificial insemination outcomes may have major economic implications because this assisted reproductive technology is the main tool for pig gene dissemination worldwide^40^.

Several mechanisms are involved in the ROS scavenging properties of H_2_S. One of these mechanisms involves H_2_S itself by virtue of its reducing properties^13^. In this way, the total antioxidant capacities of Na_2_S and GYY4137 were tested in this study based on the compounds’ abilities to reduce 2,2′-azino-bis(3-ethylbenz-thiazoline-6-sulfonic acid) (ABTS) previously oxidised with H_2_O_2_^41^. Another mechanism that may explain the capacity of H_2_S donors to palliate the damages provoked by oxidative stress concerns the enhancement of the cellular antioxidant defences. Previous studies found that H_2_S increases glutathione synthesis, decreases ROS production, and stimulates the activities of superoxide dismutase, glutathione peroxidase, and glutathione reductase^4,9,13,25,26^. Moreover, H_2_S decreases the apoptosis rate, increases the protein expression ratio of Bax/Bcl-2, and stimulates Cyp19 gene expression, among other effects^4,18,42^. Because spermatozoa are transcriptionally inactive^43^, it is likely that H_2_S donors protect sperm cells against ROS damage through their proper reducing activity, as well as by increasing the ratio of reduced to oxidised glutathione and stimulating superoxide dismutase and glutathione peroxidase activities, which represent the major antioxidant defence system of sperm cells^44^. Further studies are nevertheless required to investigate the mechanisms of action of H_2_S donors in sperm cells under oxidative stress.

In conclusion, our study provides evidence about the antioxidant properties of two H_2_S donors, Na_2_S and GYY4137; this evidence will be useful for future studies aiming to test the antioxidant effect of this gasotransmitter. Our findings clearly show that Na_2_S has a shorter and less stable total antioxidant capacity than that of GYY4137; it is even undetectable by spectrophotometry at 150 µM. However, it is important to stress out that the antioxidant capacity of GYY4137 tends to increase over time. We also found that both H_2_S donors preserve sperm motility and protect the acrosomal status against the detrimental consequences of oxidative stress, although the effects were clearly both dose and donor dependent. Within the range of concentrations tested (3–300 µM), GYY4137 showed positive effects on sperm motility, whereas Na_2_S was detrimental at the highest concentration but beneficial at the lowest. Taken together, our results suggest that Na_2_S and GYY4137 may be used as *in vitro* therapeutic agents against oxidative stress in sperm cells, although the optimal therapeutic range varies between H_2_S donors.

## Methods

Reagents were purchased from Sigma–Aldrich (Prague, Czech Republic), unless otherwise indicated.

### Ethics statement

This study did not involve animal handling because the sperm samples were purchased as artificial insemination doses from a pig breeding company (Chovservis, Hradec Králové, Czech Republic).

### Experiment I

This experiment was designed to evaluate the total antioxidant capacity and stability of Na_2_S and GYY4137 at 20, 120, and 210 minutes during incubation at 38 °C in a water bath. The solutions were prepared shortly before the experiment and kept in microcentrifuge tubes tightly sealed with the attached cap (certified free of DNA, DNase, RNase, and endotoxins (pyrogens); material: virgin polypropylene; volume: 600 µl; Neptune Scientific, San Diego, CA, USA) during the whole incubation. For each concentration of H_2_S donor, analyses were performed on the same tube throughout the incubation period. Moreover, each microcentrifuge tube contained the same volume (i.e. 200 µl) of H_2_S donor or phosphate-buffered saline (PBS; blank) solution. The experiment was replicated four times.

#### *H*_2_*S donor preparation*

Na_2_S (Na_2_S × 9 H_2_O) and GYY4137 (C_11_H_16_NO_2_PS_2_·C_4_H_9_NO × CH_2_Cl_2_) were prepared in PBS (pH≈7) solution at final concentrations of 2,400, 1,200, 600, 300, and 150 µM.

#### *Total antioxidant capacity of H*_2_*S donors*

The total antioxidant capacity was determined by spectrophotometry (Libra S22, Biochrom, Harvard Bioscience Company, Cambourne, United Kingdom) at 660 nm by using the method described previously^41^. The principle of this assay is based on the antioxidant’s capacity to reduce ABTS previously oxidised with H_2_O_2_. A standard curve was established by using known concentrations of 6-hydroxy-2,5,7,8-tetramethylchroman-2-carboxylic acid (Trolox). The total antioxidant capacity was expressed as Trolox equivalents (µM or mM). The assay was run in duplicate for each sample.

### Experiment II

This experiment was designed to test whether H_2_S donors protect sperm cells against the deleterious effects of oxidative stress.

#### Sample collection and experimental design

Artificial insemination doses from 18 boars of different breeds were purchased from a pig breeding company. Sperm-rich fractions were collected by the gloved-hand method, diluted with Solusem^®^ extender (AIM Worldwide, Vught, Netherlands; pH≈7), and transported to the laboratory at 17 °C. The sperm motility and morphology were then checked, and only samples with at least 75% of motile and morphologically normal sperm were used for the experiments. Sperm samples from three boars were pooled to reduce the effect of male variability and were centrifuged at 167 *g* for 3 minutes at 17 °C to remove debris and dead sperm cells. The sperm concentration was then checked by using a Bürker chamber, adjusted to 30–40 × 10^6^ spermatozoa/ml with Solusem^®^, and finally diluted 1:1 (v/v) with Solusem^®^ supplemented with 0.2% (w/v) of bovine serum albumin (BSA; ethanol-fractionated lyophilised powder). Thus, the final sperm and BSA concentrations were 15–20 × 10^6^ spermatozoa/ml and 0.1%, respectively. Sperm samples were then randomly split into eight microcentrifuge tubes tightly sealed with the attached cap (certified free of DNA, DNase, RNase, and endotoxins (pyrogens); material: virgin polypropylene; volume: 2 ml; Neptune Scientific, San Diego, CA, USA): CTR, CTR-ox, and Na_2_S or GYY4137 at 300, 30, and 3 µM under oxidative stress. Oxidative stress was induced by adding a solution composed of 0.1 mM FeSO_4_ and 0.5 mM sodium ascorbate (Fe^2+^/ascorbate) to the sperm samples. Because the effects of this ROS-generating system are clearly evident after 210 minutes of sperm incubation^19^, sperm analyses were performed at 0 hour (after 20 minutes of incubation for the CTR group only) and after 210 minutes of incubation at 38 °C in a water bath. The experiment was replicated six times with six independent semen pools.

#### Sperm motility

Sperm motility was evaluated by using CASA (NIS-Elements; Nikon, Tokyo, Japan, and Laboratory Imaging, Prague, Czech Republic), after loading 5 µl of sperm sample into a pre-warmed (38 °C) Spermtrack chamber (PROiSER R + D S.L., Paterna, Spain; chamber depth: 20 µm). A total of ten sperm kinetic parameters were obtained by analysing six random fields: TM (%), PM (%),VAP (µm/s), VCL (µm/s), VSL (µm/s), ALH (μm), BCF (Hz), LIN (VSL/VCL, %), STR (VSL/VAP, %), and WOB (VAP/VCL, %). The settings parameters were as follows: frames per second, 60; minimum frames acquired, 31; VAP ≥ 10 μm/s to classify a spermatozoon as motile, STR ≥ 80% to classify a spermatozoon as progressive^19^. A minimum of 200 sperm cells were analysed for each sample.

#### Sperm mitochondrial status

Mitochondrial status was evaluated as previously described^45^, with minor modifications. Briefly, aliquots of sperm samples were incubated with rhodamine 123 (5 mg/ml, w/v, in dimethyl sulfoxide, DMSO) and propidium iodide (0.5 mg/ml, w/v, in PBS) for 15 minutes at 38 °C in the dark. Subsequently, samples were centrifuged at 500 *g* for 5 minutes, the supernatant was removed, and the sperm pellet was resuspended in PBS. Then, 200 spermatozoa were evaluated by using epifluorescence microscopy (40× objective; Nikon Eclipse E600, Nikon, Tokyo, Japan): the spermatozoa showing bright green fluorescence in the midpiece were considered to have active mitochondria.

#### Sperm plasma membrane integrity

The sperm plasma membrane integrity was evaluated as previously described^46,47^. Aliquots of sperm samples were incubated with carboxyfluorescein diacetate (0.46 mg/ml, w/v, in DMSO), propidium iodide (0.5 mg/ml, w/v, in PBS), and formaldehyde solution (0.3%, v/v) for 10 minutes at 38 °C in the dark. Then, 200 spermatozoa were evaluated by using epifluorescence microscopy (40× objective). The spermatozoa showing green fluorescence over the entire head area were considered to have intact plasma membrane.

#### Lipid peroxidation

Lipid peroxidation was assessed with the thiobarbituric acid reactive substances (TBARS) assay, as previously described^19,48^. At the end of each incubation period, sperm aliquots were collected and stored at −80 °C until analysis. The absorbance of each sample was then measured by spectrophotometry at 532 nm. A standard curve was established by using known concentrations of 1,1,3,3-tetramethoxypropane (MDA). The levels of lipid peroxidation are shown as µmol of MDA per 10^8^ spermatozoa. The assay was run in duplicate for each sample.

#### Acrosomal status

Acrosome integrity was assessed after sample fixation in 2% (v/v) glutaraldehyde solution and by examination with phase-contrast microscopy (40× objective)^34^. For each sample, 200 spermatozoa were evaluated, and the percentage of sperm cells with NAR was determined. Acrosome loss was evaluated according to the protocol previously described^49^. Briefly, after methanol fixation and double washing with PBS, the samples were incubated with PNA-FITC (100 µg/ml, w/v, in PBS) for 10 minutes at 38 °C in the dark. Epifluorescence microscopy (40× objective) was used to evaluate 200 spermatozoa, and the cells showing no fluorescence over the acrosome were considered as acrosome-lost spermatozoa.

### Statistical analysis

Data were analysed with the statistical program SPSS, version 20 (IBM Inc., Chicago, IL, USA). Shapiro-Wilk’s and Levene’s tests were used to analyse the normal distribution and the variance homogeneity of the data, respectively. The Mann–Whitney U-test was applied to check for differences between the total antioxidant capacities of Na_2_S and GYY4137 at the same concentration, whereas the repeated-measures Friedman test was used to compare the total antioxidant capacities of the H_2_S donors across the incubation times. The generalized linear model (GZLM) was performed to analyse the effects of the type and concentration of H_2_S donor on the sperm variables. The statistical significance was determined at *p* < 0.05. Data are shown as the mean±standard error.

## Supplementary information

Dataset 1.

Dataset 2.

Dataset 3.

## Data Availability

All data generated or analysed during this study are included in this article and its supplementary information file.
