# Supplementary material for: Divergent effect of fast- and slow-releasing H2S donors on boar spermatozoa under oxidative stress
Source: Sci Rep. 2020 Apr 16;10:6508. doi: 10.1038/s41598-020-63489-4 (PMC7162918; doi:10.1038/s41598-020-63489-4)
Supplement: Supplementary file 1 — Dataset 1. [file 41598_2020_63489_MOESM1_ESM.pdf]

Divergent effect of fast- and slow-releasing H_2S donors on boar spermatozoa under oxidative stress

Eliana Pintus, Marija Jovičić, Martin Kadlec, José Luis Ros-Santaella

Dataset 1. Total antioxidant capacity of the H_2S donors Na_2S and GYY4137.

Treatment	Replicate	Time (minutes)	Trolox equivalents (µM)
Na_2S-2.4 mM	1	20	2236.742
Na_2S-1.2 mM	1	20	982.955
Na_2S-0.6 mM	1	20	274.621
Na_2S-0.3 mM	1	20	-32.197
Na_2S-0.15 mM	1	20	-225.379
GYY4137-2.4 mM	1	20	2662.879
GYY4137-1.2 mM	1	20	1589.015
GYY4137-0.6 mM	1	20	846.591
GYY4137-0.3 mM	1	20	337.121
GYY4137-0.15 mM	1	20	51.136
PBS	1	20	-251.894
Na_2S-2.4 mM	1	120	2223.485
Na_2S-1.2 mM	1	120	568.182
Na_2S-0.6 mM	1	120	145.833
Na_2S-0.3 mM	1	120	-39.773
Na_2S-0.15 mM	1	120	-263.258
GYY4137-2.4 mM	1	120	2729.167
GYY4137-1.2 mM	1	120	1664.773
GYY4137-0.6 mM	1	120	865.530
GYY4137-0.3 mM	1	120	344.697
GYY4137-0.15 mM	1	120	113.636
PBS	1	120	-172.348
Na_2S-2.4 mM	1	210	2113.636
Na_2S-1.2 mM	1	210	325.758
Na_2S-0.6 mM	1	210	3.788
Na_2S-0.3 mM	1	210	-162.879
Na_2S-0.15 mM	1	210	-214.015
GYY4137-2.4 mM	1	210	2765.152
GYY4137-1.2 mM	1	210	1691.288
GYY4137-0.6 mM	1	210	884.470
GYY4137-0.3 mM	1	210	346.591
GYY4137-0.15 mM	1	210	89.015
PBS	1	210	-221.591
Na_2S-2.4 mM	2	20	2649.639

Na_2S-1.2 mM	2	20	1252.048
Na_2S-0.6 mM	2	20	473.735
Na_2S-0.3 mM	2	20	220.723
Na_2S-0.15 mM	2	20	18.313
GYY4137-2.4 mM	2	20	3596.627
GYY4137-1.2 mM	2	20	2264.096
GYY4137-0.6 mM	2	20	1295.422
GYY4137-0.3 mM	2	20	695.422
GYY4137-0.15 mM	2	20	396.627
PBS	2	20	-51.566
Na_2S-2.4 mM	2	120	2372.530
Na_2S-1.2 mM	2	120	1011.084
Na_2S-0.6 mM	2	120	471.325
Na_2S-0.3 mM	2	120	242.410
Na_2S-0.15 mM	2	120	109.880
GYY4137-2.4 mM	2	120	3652.048
GYY4137-1.2 mM	2	120	2365.301
GYY4137-0.6 mM	2	120	1406.265
GYY4137-0.3 mM	2	120	803.855
GYY4137-0.15 mM	2	120	408.675
PBS	2	120	40.000
Na_2S-2.4 mM	2	210	1880.964
Na_2S-1.2 mM	2	210	799.036
Na_2S-0.6 mM	2	210	350.843
Na_2S-0.3 mM	2	210	220.723
Na_2S-0.15 mM	2	210	119.518
GYY4137-2.4 mM	2	210	3738.795
GYY4137-1.2 mM	2	210	2476.145
GYY4137-0.6 mM	2	210	1437.590
GYY4137-0.3 mM	2	210	827.952
GYY4137-0.15 mM	2	210	531.566
PBS	2	210	100.241
Na_2S-2.4 mM	3	20	2444.315
Na_2S-1.2 mM	3	20	1144.354
Na_2S-0.6 mM	3	20	445.867
Na_2S-0.3 mM	3	20	53.939
Na_2S-0.15 mM	3	20	-79.938
GYY4137-2.4 mM	3	20	2748.933
GYY4137-1.2 mM	3	20	1747.769
GYY4137-0.6 mM	3	20	927.047
GYY4137-0.3 mM	3	20	420.644
GYY4137-0.15 mM	3	20	133.489
PBS	3	20	-136.205

Na_2S-2.4 mM	3	120	2054.327
Na_2S-1.2 mM	3	120	438.106
Na_2S-0.6 mM	3	120	174.234
Na_2S-0.3 mM	3	120	-122.623
Na_2S-0.15 mM	3	120	-112.922
GYY4137-2.4 mM	3	120	2812.961
GYY4137-1.2 mM	3	120	1565.386
GYY4137-0.6 mM	3	120	812.573
GYY4137-0.3 mM	3	120	377.959
GYY4137-0.15 mM	3	120	133.489
PBS	3	120	-163.368
Na_2S-2.4 mM	3	210	1866.123
Na_2S-1.2 mM	3	210	183.935
Na_2S-0.6 mM	3	210	38.417
Na_2S-0.3 mM	3	210	-23.671
Na_2S-0.15 mM	3	210	-120.683
GYY4137-2.4 mM	3	210	2814.901
GYY4137-1.2 mM	3	210	1751.649
GYY4137-0.6 mM	3	210	1010.477
GYY4137-0.3 mM	3	210	509.895
GYY4137-0.15 mM	3	210	280.947
PBS	3	210	-149.787
Na_2S-2.4 mM	4	20	2567.878
Na_2S-1.2 mM	4	20	1333.015
Na_2S-0.6 mM	4	20	587.317
Na_2S-0.3 mM	4	20	179.414
Na_2S-0.15 mM	4	20	31.230
GYY4137-2.4 mM	4	20	2375.080
GYY4137-1.2 mM	4	20	1379.222
GYY4137-0.6 mM	4	20	764.181
GYY4137-0.3 mM	4	20	370.618
GYY4137-0.15 mM	4	20	195.347
PBS	4	20	-16.571
Na_2S-2.4 mM	4	120	2398.980
Na_2S-1.2 mM	4	120	1132.250
Na_2S-0.6 mM	4	120	322.817
Na_2S-0.3 mM	4	120	98.152
Na_2S-0.15 mM	4	120	0.956
GYY4137-2.4 mM	4	120	2461.122
GYY4137-1.2 mM	4	120	1508.286
GYY4137-0.6 mM	4	120	966.539
GYY4137-0.3 mM	4	120	604.844
GYY4137-0.15 mM	4	120	302.103

PBS	4	120	59.911
Na_2S-2.4 mM	4	210	2247.610
Na_2S-1.2 mM	4	210	992.033
Na_2S-0.6 mM	4	210	247.929
Na_2S-0.3 mM	4	210	136.393
Na_2S-0.15 mM	4	210	88.591
GYY4137-2.4 mM	4	210	2497.769
GYY4137-1.2 mM	4	210	1549.713
GYY4137-0.6 mM	4	210	945.825
GYY4137-0.3 mM	4	210	499.681
GYY4137-0.15 mM	4	210	255.895
PBS	4	210	133.206

PBS: phosphate-buffered saline solution.
