# Supplementary material for: Divergent effect of fast- and slow-releasing H2S donors on boar spermatozoa under oxidative stress
Source: Sci Rep. 2020 Apr 16;10:6508. doi: 10.1038/s41598-020-63489-4 (PMC7162918; doi:10.1038/s41598-020-63489-4)
Supplement: Supplementary file 2 — Dataset 2. [file 41598_2020_63489_MOESM2_ESM.pdf]

Divergent effect of fast- and slow-releasing H_2S donors on boar spermatozoa under oxidative stress

Eliana Pintus, Marija Jovičić, Martin Kadlec, José Luis Ros-Santaella

Dataset 2. Boar sperm motility under induced oxidative stress (except for CTR) and supplemented with the H_2S donors Na_2S and GYY4137.

Treatment	Replicate	Time (min)	TM (%)	PM (%)	VAP (µm/s)	VCL (µm/s)	VSL (µm/s)	ALH (µm)	BCF (Hz)	LIN (%)	STR (%)	WOB (%)
CTR	1	0	82.711	56.63	46.035	88.272	37.517	3.439	13.175	42.606	80.917	51.218
CTR	1	210	82.455	60.346	54.583	91.720	44.545	6.047	13.530	49.870	81.034	60.421
CTR-ox	1	210	76.219	69.547	46.103	72.129	40.926	3.010	16.525	59.507	89.341	65.752
GYY4137-ox-300µM	1	210	75.912	64.152	48.422	84.209	42.045	3.352	15.036	50.910	85.742	58.409
GYY4137-ox-30µM	1	210	87.733	75.534	47.503	79.331	42.039	3.339	15.313	54.008	87.856	60.574
GYY4137-ox-3µM	1	210	85.694	76.983	47.337	79.953	42.496	3.108	15.835	54.073	88.890	60.009
Na_2S-ox-300µM	1	210										
Na_2S-ox-30µM	1	210	3.830	55.83	19.030	32.239	18.001	1.262	15.252	61.528	94.282	64.463
Na_2S-ox-3µM	1	210	79.810	81.34	35.564	57.016	33.251	2.308	15.929	59.242	92.518	63.339
CTR	2	0	62.649	47.72	38.379	73.833	32.318	2.685	13.940	43.605	83.425	51.055
CTR	2	210	64.320	59.36	37.681	65.504	32.403	2.605	15.112	51.413	86.794	58.064
CTR-ox	2	210	38.710	60.945	23.298	34.417	22.251	1.429	16.911	65.413	94.970	68.517
GYY4137-ox-300µM	2	210	48.092	69.478	23.685	36.284	22.403	1.581	15.690	62.417	93.951	66.008
GYY4137-ox-30µM	2	210	61.094	74.343	28.217	43.281	26.601	1.891	16.197	62.367	93.623	66.157
GYY4137-ox-3µM	2	210	51.979	63.47	32.756	53.342	30.070	2.543	15.774	56.812	90.852	61.995
Na_2S-ox-300µM	2	210										
Na_2S-ox-30µM	2	210	3.511	40.426	19.363	31.954	17.589	1.280	13.650	56.730	91.828	61.508
Na_2S-ox-3µM	2	210	66.161	56.872	42.605	72.221	35.519	2.770	15.914	50.228	82.866	59.607
CTR	3	0	80.624	78.383	43.026	73.281	39.668	2.800	15.089	52.805	91.791	56.937
CTR	3	210	80.512	85.993	39.484	54.321	38.074	2.281	17.988	68.020	95.593	70.673
CTR-ox	3	210	59.884	66.936	38.625	67.214	36.291	2.584	15.117	56.527	92.097	59.680

GYY4137-ox-300µM	3	210	76.362	85.2	40.406	54.549	38.947	2.482	16.678	71.132	95.808	73.834
GYY4137-ox-30µM	3	210	80.244	87.271	41.283	57.478	39.807	2.535	17.140	69.389	95.937	71.957
GYY4137-ox-3µM	3	210	84.534	88.559	48.797	70.661	46.708	3.096	16.288	65.575	95.062	68.588
Na_2S-ox-300µM	3	210										
Na_2S-ox-30µM	3	210	1.176	30.42	20.520	33.152	19.700	1.406	16.321	63.811	96.143	66.257
Na_2S-ox-3µM	3	210	74.718	83.46	41.000	55.238	39.746	2.489	17.197	70.793	96.274	73.116
CTR	4	0	73.039	37.772	39.533	88.894	28.756	3.100	11.731	32.128	72.242	42.502
CTR	4	210	61.902	58.91	39.111	72.451	34.668	2.717	14.948	49.069	88.660	54.135
CTR-ox	4	210	41.818	59.056	27.696	44.182	26.387	1.769	15.859	62.201	94.601	64.947
GYY4137-ox-300µM	4	210	44.077	60.769	28.059	46.368	26.648	1.895	15.682	59.674	94.403	62.536
GYY4137-ox-30µM	4	210	63.333	70	37.011	62.949	33.783	2.546	14.970	55.079	90.733	59.734
GYY4137-ox-3µM	4	210	78.919	69.421	49.327	86.573	42.844	3.337	15.130	50.483	86.754	57.058
Na_2S-ox-300µM	4	210										
Na_2S-ox-30µM	4	210	0.935	38.902	19.859	30.838	17.982	1.467	14.595	66.004	94.484	69.308
Na_2S-ox-3µM	4	210	75.540	52.998	48.965	100.782	38.961	3.442	13.918	41.317	81.014	49.526
CTR	5	0	78.515	53.862	41.833	82.498	34.369	2.954	13.282	40.658	81.202	48.676
CTR	5	210	76.570	79.86	38.745	63.700	36.673	2.446	15.490	56.343	93.355	59.565
CTR-ox	5	210	50.407	64.36	47.590	78.493	44.461	3.222	15.242	58.199	92.258	62.290
GYY4137-ox-300µM	5	210	61.747	70.602	46.364	76.769	42.868	3.168	15.400	57.826	91.430	62.085
GYY4137-ox-30µM	5	210	76.117	81.65	47.724	73.438	45.023	3.185	15.828	62.287	93.678	65.920
GYY4137-ox-3µM	5	210	77.429	79.624	43.892	69.016	41.126	2.889	15.373	60.321	92.866	64.227
Na_2S-ox-300µM	5	210										
Na_2S-ox-30µM	5	210	29.817	59.212	29.822	45.549	28.462	1.949	15.738	63.364	94.830	66.255
Na_2S-ox-3µM	5	210	58.258	74.473	36.302	57.642	34.739	2.477	14.879	60.748	94.749	63.630
CTR	6	0	76.173	41.651	49.671	96.013	36.128	3.339	13.703	38.589	74.568	50.257
CTR	6	210	72.962	81.198	42.400	66.244	40.355	2.564	16.392	59.921	94.161	63.073
CTR-ox	6	210	12.908	74.481	15.003	19.676	14.628	0.837	18.233	75.655	97.569	77.486
GYY4137-ox-300µM	6	210	36.759	72.859	26.171	37.873	25.371	1.538	17.256	68.668	96.600	70.789
GYY4137-ox-30µM	6	210	45.568	74.244	27.100	37.545	26.100	1.567	17.693	70.267	95.974	72.893
GYY4137-ox-3µM	6	210	40.389	73.570	25.559	36.083	24.723	1.474	18.406	68.806	96.261	71.175

Na_2S-ox-300µM	6	210										
Na_2S-ox-30µM	6	210										
Na_2S-ox-3µM	6	210	39.096	68.484	30.537	45.035	29.392	1.815	16.585	66.250	95.997	68.796

TM: total motility; PM: progressive motility; VAP: average path velocity; VCL: curvilinear velocity; VSL: straight-line velocity; ALH: amplitude of lateral head displacement; BCF: beat-cross frequency; LIN: linearity (VSL/VCL); STR: straightness (VSL/VAP); WOB: wobble (VAP/VCL); CTR: control; ox: samples submitted to induced oxidative stress.
