# Supplementary material for: Divergent effect of fast- and slow-releasing H2S donors on boar spermatozoa under oxidative stress
Source: Sci Rep. 2020 Apr 16;10:6508. doi: 10.1038/s41598-020-63489-4 (PMC7162918; doi:10.1038/s41598-020-63489-4)
Supplement: Supplementary file 3 — Dataset 3. [file 41598_2020_63489_MOESM3_ESM.pdf]

Divergent effect of fast- and slow-releasing H_2S donors on boar spermatozoa under oxidative stress

Eliana Pintus, Marija Jovičić, Martin Kadlec, José Luis Ros-Santaella

Dataset 3. Boar sperm mitochondrial status, plasma membrane integrity, acrosomal status, and lipid peroxidation under induced oxidative stress (except for CTR) and supplemented with the H_2S donors Na_2S and GYY4137.

Treatment	Replicate	Time (min)	AM (%)	IPM (%)	NAR (%)	AL (PNA%)	LP (µmol MDA/10^8 sptz)
CTR	1	0	55.5	80.0	92.0	2.0	5.858
CTR	1	210	67.5	79.0	96.0	3.0	5.858
CTR-ox	1	210	63.0	72.0	90.5	6.0	47.769
GYY4137-ox-300µM	1	210	65.5	77.0	92.5	3.5	44.840
GYY4137-ox-30µM	1	210	59.5	78.0	95.0	3.0	49.685
GYY4137-ox-3µM	1	210	65.0	74.0	94.5	3.0	47.431
Na_2S-ox-300µM	1	210	0.0	26.5	19.5	3.0	62.528
Na_2S-ox-30µM	1	210	74.0	74.5	93.5	2.5	51.149
Na_2S-ox-3µM	1	210	60.5	76.5	91.5	3.0	50.699
CTR	2	0	62.5	85.5	94.5	2.5	2.817
CTR	2	210	70.5	82.0	91.5	3.0	3.831
CTR-ox	2	210	60.0	73.5	90.5	5.0	44.051
GYY4137-ox-300µM	2	210	63.5	78.0	89.0	2.0	40.108
GYY4137-ox-30µM	2	210	75.5	80.0	93.5	3.0	44.164
GYY4137-ox-3µM	2	210	57.5	79.0	95.0	2.5	42.699
Na_2S-ox-300µM	2	210	0.0	13.0	52.5	2.5	50.924
Na_2S-ox-30µM	2	210	65.5	72.5	96.5	3.5	44.389
Na_2S-ox-3µM	2	210	70.5	76.0	93.5	2.5	44.051
CTR	3	0	46.0	80.5	96.5	0.5	3.943
CTR	3	210	62.0	75.5	92.5	2.0	3.943
CTR-ox	3	210	61.5	58.0	94.0	2.5	45.967
GYY4137-ox-300µM	3	210	66.5	62.0	97.0	0.5	43.826
GYY4137-ox-30µM	3	210	62.0	69.0	96.5	1.5	43.150
GYY4137-ox-3µM	3	210	58.5	69.5	93.5	2.0	42.136
Na_2S-ox-300µM	3	210	0.0	9.0	22.5	2.0	52.163

Na_2S-ox-30µM	3	210	51.5	56.0	96.0	2.5	44.164
Na_2S-ox-3µM	3	210	51.5	65.0	95.0	1.0	42.812
CTR	4	0	60.5	86.0	95.5	0.5	2.479
CTR	4	210	70.0	75.0	98.0	2.5	4.281
CTR-ox	4	210	62.0	77.5	93.5	2.5	44.840
GYY4137-ox-300µM	4	210	70.0	73.0	97.0	1.0	42.023
GYY4137-ox-30µM	4	210	66.0	79.5	96.0	1.0	41.347
GYY4137-ox-3µM	4	210	68.0	85.0	95.5	0.5	42.249
Na_2S-ox-300µM	4	210	0.0	34.5	19.5	1.0	52.839
Na_2S-ox-30µM	4	210	60.5	72.0	96.0	1.0	43.263
Na_2S-ox-3µM	4	210	61.0	80.0	93.0	1.0	41.460
CTR	5	0	64.5	87.0	98.0	1.5	3.380
CTR	5	210	56.0	81.5	95.5	2.5	7.210
CTR-ox	5	210	68.0	72.0	95.0	2.5	38.869
GYY4137-ox-300µM	5	210	80.0	78.5	90.0	1.0	37.968
GYY4137-ox-30µM	5	210	59.5	84.5	91.0	3.0	38.982
GYY4137-ox-3µM	5	210	67.5	87.0	95.0	2.0	37.630
Na_2S-ox-300µM	5	210	0.0	40.0	30.0	1.5	45.629
Na_2S-ox-30µM	5	210	72.0	74.0	93.0	2.0	39.320
Na_2S-ox-3µM	5	210	72.5	85.5	91.0	1.5	41.573
CTR	6	0	47.0	73.0	94.0	1.5	2.748
CTR	6	210	51.0	66.5	95.0	1.5	2.748
CTR-ox	6	210	60.0	54.0	94.0	5.0	29.889
GYY4137-ox-300µM	6	210	60.5	56.5	92.5	1.0	25.914
GYY4137-ox-30µM	6	210	55.0	63.5	92.5	2.0	30.570
GYY4137-ox-3µM	6	210	62.0	64.5	91.5	1.0	32.387
Na_2S-ox-300µM	6	210	0.0	4.0	42.5	2.0	39.655
Na_2S-ox-30µM	6	210	41.5	47.5	92.5	1.5	32.273
Na_2S-ox-3µM	6	210	56.5	66.0	92.5	1.0	28.640

AM: active mitochondria; IPM: intact plasma membrane; NAR: normal apical ridge; AL: acrosome loss; PNA: peanut agglutinin-fluorescein isothiocyanate; LP: lipid peroxidation; MDA: malondialdehyde; sptz: spermatozoa. CTR: control; ox: samples submitted to induced oxidative stress.
